# Ferrocene-Modified Polyacrylonitrile-Containing Block Copolymers as Preceramic Materials

**DOI:** 10.3390/polym16152142

**Published:** 2024-07-28

**Authors:** Sebastian Heinz, Lea Gemmer, Oliver Janka, Markus Gallei

**Affiliations:** 1 Polymer Chemistry, Campus C4 2, Saarland University, 66123 Saarbrücken, Germany; sebastian.heinz@uni-saarland.de (S.H.); lea.gemmer@tu-darmstadt.de (L.G.); 2 Inorganic Solid State Chemistry, Campus C4 1, Saarland University, 66123 Saarbrücken, Germany; oliver.janka@uni-saarland.de; 3 Saarene, Campus C4 2, Saarland Center for Energy Materials and Sustainability, 66123 Saarbrücken, Germany

**Keywords:** polyacrylonitrile, block copolymers, carbon materials, ferrocene, metallopolymers

## Abstract

In the pursuit of fabricating functional ceramic nanostructures, the design of preceramic functional polymers has garnered significant interest. With their easily adaptable chemical composition, molecular structure, and processing versatility, these polymers hold immense potential in this field. Our study succeeded in focusing on synthesizing ferrocene-containing block copolymers (BCPs) based on polyacrylonitrile (PAN). The synthesis is accomplished via different poly(acrylonitrile-*block*-methacrylate)s via atom transfer radical polymerization (ATRP) and activators regenerated by electron transfer ATRP (ARGET ATRP) for the PAN macroinitiators. The molecular weights of the BCPs range from 44 to 82 kDa with dispersities between 1.19 and 1.5 as determined by SEC measurements. The volume fraction of the PMMA block ranges from 0.16 to 0.75 as determined by NMR. The post-modification of the BCPs using 3-ferrocenyl propylamine has led to the creation of redox-responsive preceramic polymers. The thermal stabilization of the polymer film has resulted in stabilized morphologies based on the oxidative PAN chemistry. The final pyrolysis of the sacrificial block segment and conversion of the metallopolymer has led to the formation of a porous carbon network with an iron oxide functionalized surface, investigated by scanning electron microscopy (SEM), energy dispersive X-ray mapping (EDX), and powder X-ray diffraction (PXRD). These findings could have significant implications in various applications, demonstrating the practical value of our research in convenient ceramic material design.

## 1. Introduction

With their unique self-organization capabilities, block copolymers (BCPs) hold immense potential in various research fields. Their diverse and intriguing applications range from nanolithography and photonic materials to drug delivery and separation technologies [1,2,3]. The current focus in nanotechnology is on developing multi-dimensional materials and fundamental concepts for the tailored design of functional materials based on BCPs [4,5]. In general, BCPs consisting of two or more covalently connected polymer segments are capable of self-organization, also known as microphase separation [6,7]. They are excellent materials for creating tailor-made nanostructures in the bulk state or selective solvents [8,9,10,11]. The resulting structures can provide different responses based on functional moieties as part of the underlying block segments [12]. Structural changes may influence optical, electrical, magnetic, and chemical properties. Moreover, different templating strategies based on BCPs have been applied as a feasible preparation route to control the shape and size of the final materials after removing the template structure [13,14,15,16]. Carbon-based (nano)materials have sparked enormous interest due to their exciting properties and potential applications in various fields like batteries, supercapacitors, or biomedicine [17]. However, the journey toward industrial-scale porous carbon materials is challenging. The typical multi-step procedures often result in fragile structures with defects, posing a significant hurdle. These challenges underline the need for further research and development, as the potential of carbon-based materials remains inspiring, and their applications continue to expand. Preparing carbonaceous materials and combinations with other moieties and functionalities based on a single-source precursor avoids additional infiltration steps. It is a promising technique for preparing ordered porous materials using BCPs and particle architectures. Poly(acrylonitrile) (PAN), BCPs thereof, and polymer particle architectures were reported as suitable carbon precursor materials with tailored porosity [18,19,20,21,22,23,24,25,26]. PAN-derived carbon materials attracted much attention in catalytic applications, such as absorbers, membranes, or electrode materials in batteries [27,28,29,30]. Thermally-induced stabilization of PAN or copolymers is an interesting strategy for producing PAN-derived carbon materials. The oxidative stabilization of PAN typically occurs at temperatures above 200 °C under air, leading to a change of the chemical structure comprising inter- and intramolecular cyclization reactions [30,31]. Further pyrolysis of such stabilized PAN-containing BCPs enables cleavage of a sacrificial block segment while maintaining the structure of the stabilized PAN block. PAN is a rigid and solvent-resistant material because of the small size of the acrylonitrile moiety and its polar nature [32,33]. Acrylonitrile is used as a comonomer to increase commercial polymers’ mechanical stability and solvent resistance [31,34]. Tailoring the macromolecular chain architecture to control polymerization strategies would be favorable and acrylonitrile has been subject to some controlled polymerization methods nowadays [35,36,37,38,39,40,41,42,43,44]. However, the controlled polymerization of acrylonitrile has mainly caused issues for classical controlled radical polymerization techniques, for example, the atom transfer radical polymerization (ATRP), as side reactions with the copper catalyst occur [42,45]. This resulted in limitations in achievable molecular weights of PAN [42,46] and corresponding copolymers with styrene (PSAN) [47,48]. The re-initiation capability of the first block segment for adding a second monomer to achieve BCPs is, therefore, coming with issues.

Besides the PAN’s ability to form carbon materials upon heating, other functional polymer segments are interesting. Within this contribution, we are aiming for the tailored design of PAN-based BCPs and the class of metallopolymers to produce carbon/iron ceramic after thermal treatment of tailored BCPs for the first time. In general, polymers can feature metal centers as part of their backbone or side chains. Within this field, so-called metallopolymers attracted enormous attention because of their excellent combination of redox-mediated switching capabilities, improved mechanical properties, semi-conductivity, photophysical, optoelectronic, and catalytic properties, as well as separation capabilities [49,50,51,52,53,54,55,56,57,58]. The readers are referred to reviews in the field of main-chain and side-chain-containing metallopolymers given by Zhou et al. [59], the Tang group [60,61], and other authors [52,62]. Using metallopolymers as preceramic materials is of great interest, as iron-containing polymers can generate iron oxide-based nanocomposites [63,64,65]. Despite using metal-containing monomers such as vinyl ferrocene or ferrocenyl methacrylate to prepare metallopolymers, the metal functionality can also be introduced by the post-functionalization of metallocene segments [66].

This contribution presents concepts for synthesizing well-defined polymer-templated inorganic carbon and carbon/iron oxide structures with hierarchical nano/microstructures. For this purpose, PAN-*b*-poly(methyl methacrylate) (PMMA) BCPs are synthesized via ATRP and investigated concerning their molecular characteristics. Stabilization and pyrolysis of the BCP films enable the formation of porous structures and lamella morphologies. In addition, post-modification protocols for the BCPs are advantageously used to tune the properties and structure of the resulting carbon material. At the same time, partial amidation of the PMMA block segment with 3-ferrocenyl propylamine is shown to introduce other components for mixed metal oxides within the porous carbon material, generating new interesting carbon/metal composite materials. Such materials could be further investigated regarding their application in catalytic processes or other applications where a porous material is needed. Furthermore, this work shows a convenient approach for the modification of PAN-based carbon materials, which could be easily modified with other metals using the same method.

## 2. Materials and Methods

In general PAN-macro initiators were prepared by copper-mediated ARGET-ATRP. This is followed by a classical ATRP of MMA using the previously synthesized PAN as a macro initiator, yielding the corresponding BCPs. A partial amidation of the PMMA segment of the polymer with 3-ferrocenyl propylamine enabled the block-selective post-modification of the BCP. The subsequent treatment of the polymers involved stabilization and pyrolysis in order to yield porous carbon or carbon/iron ceramic structures. All syntheses were performed under an argon atmosphere with baked-out glassware.

### 2.1. Reagents

Methyl methacrylate (MMA, 99%), tin(II) 2-ethylhexanoate (Sn(EH)_2_, 92.5–100%), tris(2-pyridylmethyl)amine (TPMA, 99%), CuCl_2_ (99.999%), CuBr (99.999%), 2-bromopropionitrile (BPN, 99%), 2,2’-bipyridine (BPY, 99%), and ferrocene carboxyaldehyde (98%) were purchased from Sigma Aldrich (St. Louis, MA, USA). Raney Ni (50 wt.-% in water) was purchased from ABCR (Karlsruhe, Germany). Acrylonitrile (AN, 99%) was purchased from TCI (Tokyo, Japan). All other chemicals were purchased from Carl Roth (Karlsruhe, Germany), Sigma Aldrich, TCI, and Fisher Scientific (Schwerte, Germany). All chemicals were used as received, if not stated otherwise. Before polymerization, inhibitors from the monomers AN and MMA were removed by passing over a basic alumina column. AN was additionally stirred over calcium hydride, degassed by freeze–pump–thaw, and distilled under reduced pressure before being stored in the freezer inside a nitrogen-filled glovebox. Dimethylsulfoxide used for the catalyst stock solutions was stirred over calcium hydride, degassed by freeze–pump–thaw, distilled under reduced pressure, and stored in the above-mentioned glovebox. CuBr was stirred over glacial acetic acid, washed with absolute ethanol, dried under reduced pressure, and stored in the glovebox [67].

### 2.2. Instrumentations

Nuclear magnetic resonance (NMR) spectra were recorded on a Bruker Avance II 400 spectrometer (Billerica, MA, USA) with a 9.4 T Ultrashield Plus Magnet and a BBFO probe and referenced using the solvent signals. For processing and evaluation of the spectra, MestReNova 14.2.0 was used. Differential scanning calorimetry (DSC) was carried out on a Netzsch DSC 214 Polyma (Selb, Germany) with a heating rate of 10 K min^−1^ and nitrogen as protective and purge gas in flow rates of 60 mL min^−1^ and 40 mL min^−1^, respectively. For evaluation, Netzsch Proteus Thermal Analysis 8.0.1 was used. Thermal treatments were carried out using a Netzsch TG 209 F1 Libra (Selb, Germany). For evaluation, Netzsch Proteus Thermal Analysis 8.0.1 was used. Scanning electron microscope (SEM) was carried out on a Zeiss Sigma VP device (GeminiSEM 500) (Oberkochen, Germany) using the software SmartSEM Version 6.07. The samples were mounted on an aluminum stub using adhesive carbon tape and sputter coated with approximately 6 nm platinum using an Automatic Turbo Coater PLASMATOOL 125 SIN 2020_131 from Ingenieurbüro Peter Liebscher (Wetzlar, Germany). For transmission electron microscopy (TEM) analysis, ultrathin sections (40 nm) were prepared with an ultramicrotome (Reichert Ultracut by Leica Microsystems, Wetzlar, Germany) and placed on a copper grid. A thin carbon layer was added via vacuum decomposition to obtain stable films without charging during transmission electron microscopic examination. Bright-field TEM images were acquired using a JEOL JEM-2100 LaB6 electron microscope (JEOL, Tokyo, Japan) operating at 200 kV acceleration voltage; 0.14 nm lattice resolution equipped with a Gatan Orius SC1000 camera (AMETEK, Berwyn, PA, USA) (binning 2; 1024 × 1024 pixels). Standard size exclusion chromatography (SEC) with DMF as the solvent was performed with a Waters system (Milford, MA, USA) composed of a 515 HPLC Pump, a 2487 UV-detector at 260 nm and a 2410 RI-detector at 40 °C, with DMF (1 g L^−1^ LiBr) as the mobile phase (flow rate 1 mL min^−1^) on a GRAM column set (GRAM 30, GRAM 1000, GRAM 1000) from PSS (Mainz, Germany) at 60 °C. Calibration was carried out using PMMA and PEG standards. The software PSS WinGPC UniChrom V 8.31 was used for data acquisition and evaluation of the measurements. Powder X-ray diffraction (PXRD) patterns of the pulverized samples were recorded at room temperature on a D8-A25-Advance diffractometer (Bruker, Karlsruhe, Germany) in Bragg–Brentano *θ*-*θ*-geometry (goniometer radius 280 mm) with Cu Kα-radiation (*λ* = 154.0596 pm). A 12 µm Ni foil working as Kβ filter and a variable divergence slit were mounted at the primary beam side. A LYNXEYE detector with 192 channels was used at the secondary beam side. Experiments were carried out in a 2*θ* range of 7 to 120° with a step size of 0.013° and a total scan time of 2 h.

### 2.3. General Synthesis of PAN Macroinitiator

The synthesis follows a modified protocol of Dong et al. [68]. In a 100 mL Schlenk flask equipped with a stir bar and an extra stopcock, ethylene carbonate (EC) and AN were mixed and degassed by one cycle of freeze–pump–thaw. The flask was submerged again in liquid nitrogen and purged with argon. Cu(TPMA)Cl_2_ (0.05 eq., 0.2 M in DMSO) and BPN (1 eq., 0.5 M in DMSO) were added to the flask, and the rubber septum was exchanged with a glass stopper. The mixture was further degassed by two additional cycles of freeze–pump–thaw. The mixture was submerged in a pre-heated oil bath at 60 °C. To initiate the polymerization, Sn(EH)_2_/TPMA (0.5 eq., 0.1 M in DMSO) was added through the extra stopcock and sealed with a rubber septum, which was closed after the addition. The polymerization was stopped by cooling the mixture with an ice bath and opening the flask to air. The mixture was diluted with DMSO, and the polymer was precipitated in a 10-fold excess of a methanol/water mixture (90/10) by volume. After filtration, the polymer was dried under the exclusion of light in the vacuum. The polymer was analyzed via SEC.

### 2.4. General Synthesis of PAN-b-PMMA Block Copolymers

The PAN-macroinitiator was dissolved in dry DMSO in a Schlenk flask with a stir bar. MMA was added, and the mixture was degassed by three cycles of freeze–pump–thaw. The flask was purged with argon and submerged into an oil bath pre-heated to 65 °C. Cu(BPY)_2_Cl (1 eq., 0.2 M in DMSO) was added to the mixture to initiate the polymerization. After a specific reaction time, the polymerization was stopped by cooling the flask with an ice bath. The solution was diluted with DMSO, and the polymer was precipitated in a 10-fold excess of distilled water. After filtration, the polymer was dried at 40 °C under reduced pressure. The polymer was analyzed using SEC, NMR, and DSC.

### 2.5. Post-Modification of the PAN-b-PMMA Block Copolymers Using 3-Ferrocenyl Propylamine

The 3-ferrocenyl propylamine was synthesized according to a procedure by the group of Su [69]. The polymer was dissolved in DMF for post-modification, and 3-ferrocenyl propylamine was added (2 eq., respectively, to PMMA). DABCO (2 eq. respective to PMMA) was added, and the mixture was heated to 70 °C for 24 h. After cooling, the polymer was precipitated in water, filtered off, washed multiple times using water and ethanol, and dried at 40 °C under reduced pressure.

### 2.6. Stabilization and Pyrolysis of the Block Copolymer Films

To produce the block copolymer films, 30 mg of the BCPs were dissolved in 0.4 mL DMF and stirred for 3 h. The solution was filtered using a syringe with a filter, and the filter was washed with an additional 0.4 mL DMF. The vial was purged with argon, and the mixture was stirred for two days. The stir bar was removed, and the vial was put into a bigger vial halfway filled with DMF. Under a nitrogen atmosphere, the DMF slowly evaporated at 90 °C for two to three days at 110 °C. The final film was dried at 40 °C under reduced pressure. For the stabilization reaction of the PAN block, a film sample was put into the TGA and heated under synthetic air to 240 °C with a rate of 1 K min^–1^, holding the temperature for 10 h. After cooling, the subsequent pyrolysis of the material was conducted by heating the sample to 240 °C at a rate of 20 K min^–1^ and from 240 °C to 600 °C at a rate of 2 K min^–1^, holding the temperature for 5 h.

## 3. Results and Discussion

### 3.1. Synthesis and Characterization of PAN-b-PMMA Block Copolymers

**Scheme 1:**
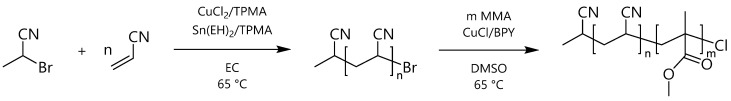
Synthesis of PAN-b-PMMA block copolymers via ARGET-ATRP and ATRP. Tris(2-pyridyl methyl)amine (TPMA); tin(II) 2-ethyl hexanoate (Sn(EH)_2_); ethylene carbonate (EC); methyl methacrylate (MMA); 2,2´-bipyridine (BPY); dimethyl sulfoxide (DMSO).

The PAN-b-PMMA BCPs in this work were synthesized by combining two different ATRP methods. The ARGET-ATRP for the polyacrylonitrile macroinitiator is used as the first block, and the second block, which consists of poly(methyl methacrylate), is added via the classical ATRP (Scheme 1). This system was chosen as it allows good control over the polymerization and overcomes synthetic issues for the controlled polymerization of acrylonitrile, as described in the introduction. Using the ARGET-ATRP for the first block of the block copolymer enables using a very small amount of catalyst. By employing Sn(EH)_2_ as a reducing agent, any Cu(II) species formed during the polymerization is reduced to Cu(I) species which catalyzes the polymerization. SEC measurements of the PAN macroinitiator against PEG standards give a relatively exact value for the molecular weight, as the hydrodynamic radius of PAN and PEG is similar (Figure S1, Table S3) [70]. Due to the very low amount of catalyst, the PAN macroinitiator still carries bromine as its end group, which enables a fast initiation of the second block. The subscripts used in PAN-*b*-PMMA block copolymers refer to the molecular weight of the corresponding block in kg mol^−1^.

**Figure 1:**
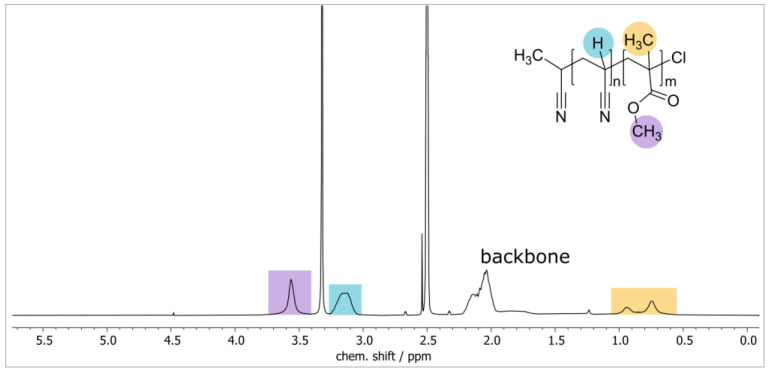
^1^H-NMR spectrum of PAN_45.7_ -*b*-PMMA*_20_*_.4_ in DMSO-d_6_.

**Figure 2:**
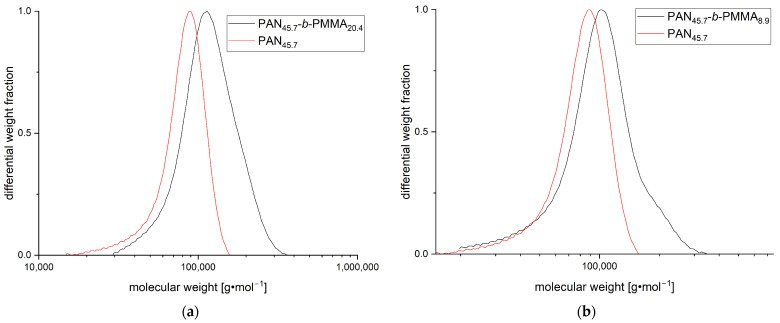
SEC measurements of the PAN macroinitiator and block copolymers (**a**) PAN_45.7_-*b*-PMMA_20.4_ and (**b**) PAN_45.7_-*b*-PMMA_8.9_, measured against PMMA standard. Measurements were performed using DMF (1 g L^−1^ LiBr) as the mobile phase.

The ^1^H-NMR-spectrum of the PAN-*b*-PMMA in Figure 1 shows the broad peaks of the methyl group of the PMMA at 3.56 ppm and the signal of the single proton of the polyacrylonitrile backbone at 3.15 ppm. Using the molecular weight of the PAN macroinitiators as determined from SEC measurements against PEG standards and the integration of the distinct signals of the two monomers from the NMR spectrum, the molecular weight of the PMMA block and, therefore, the molecular weight of the BCP could be calculated. The SEC measurements in Figure 2 display a range of the macroinitiator and the final BCP measured against PMMA standards. A shift of the BCP to higher molecular weights concerning the first block could be observed. A slight increase in the dispersity of the BCP comes in hand with a broadening of the signal (Table 1).

### 3.2. Thermal Stabilization and Pyrolysis of the Block Copolymer Films

**Figure 3:**
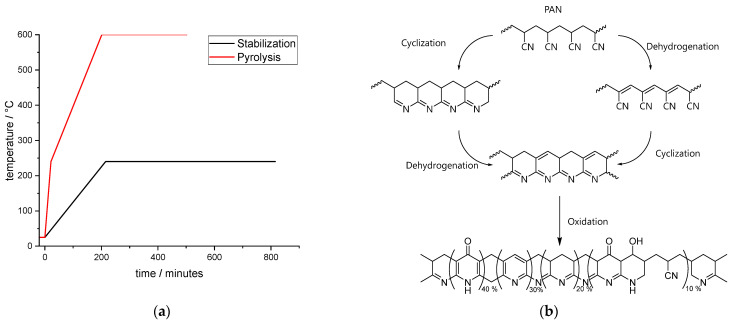
Temperature profile for the stabilization and pyrolysis of the block copolymer films. Stabilization was conducted under synthetic air, whereas the pyrolysis was achieved under nitrogen atmosphere (**a**) and a schematic example of the oxidative stabilization of polyacrylonitrile at elevated temperatures (**b**).

The thermal stabilization of the polyacrylonitrile was achieved at 240 °C under synthetic air. The temperature was maintained for 10 h to ensure the material had enough time to fully stabilize. The consecutive pyrolysis of the material was carried out at 600 °C under a nitrogen atmosphere. The thermal treatment condition was kept constant at 600 °C for 5 h. The temperature profiles of both the stabilization and the pyrolysis are shown in Figure 3a. During the stabilization, the polyacrylonitrile forms a network of six-membered ring structures through a cyclization and dehydrogenation reaction of the nitrile groups, as reported by Houtz [73]. With the change in structure during the stabilization, the morphology can be preserved and the carbonic yield is increased. Different species are formed upon further oxidation, as shown in the schematic illustration of the stabilization process in Figure 3b. This stabilization process is accompanied by a shrinkage of the domain volume. In the subsequent pyrolysis of the sample, the PMMA domain, which does not become stabilized in the previous step, decomposes, resulting in the structures shown in the SEM images. Altering the different volume fractions of the blocks, different porous or dense structures can be formed [31].

**Figure 4:**
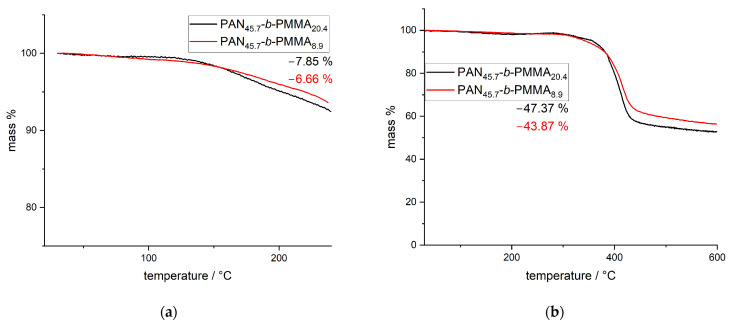
(**a**) Stabilization of the block copolymers PAN_45.7_-*b*-PMMA_20.4_ and PAN_45.7_-*b*-PMMA_8.9_ under synthetic air at 240 °C for 10 h with a heating rate of 1 K min^−1^ (**b**) and pyrolysis under nitrogen atmosphere at 600 °C for 5 h. Runs of PAN_45.7_ -*b*-PMMA_20._ and PAN_45.7_-*b*-PMMA_8.9_ conducted in the TGA. The temperature profiles for stabilization and pyrolysis followed the ones shown in Figure 3a.

**Figure 5:**
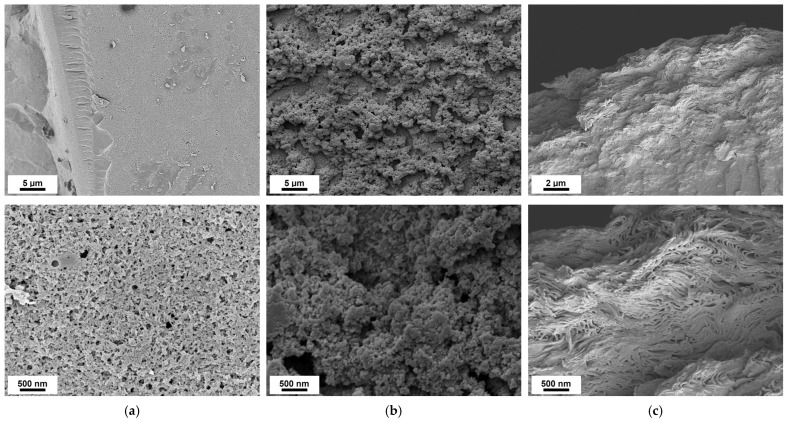
Scanning electron microscopy images of the pyrolyzed BCP film of (**a**) PAN_45.7_ -*b*-PMMA_20.4_, (**b**) PAN_45.7_-*b*-PMMA_8.9_, and (**c**) PAN_58.4_-*b*-PMMA_24.0_.

The TGA measurements of the stabilization process in Figure 4a showed that the BCPs lose about 6.5 to 8 percent of their mass during the stabilization at 240 °C for 10 h. This loss can be explained by small residues of solvent enclosed in the film and some volatile side products formed during the process [74,75]. In the pyrolysis run shown in Figure 4b, a loss of around 45 percent mass could be observed. At around 380 °C, the polymethyl methacrylate block decomposes, resulting in the observed mass loss [76]. The higher loss visible for the PAN_45.7_-*b*-PMMA_20.4_ corresponds to the larger PMMA block. The higher the PMMA content in the BCP, the higher the mass loss of the BCP during the pyrolysis step. SEM images of the pyrolyzed films show a porous surface structure (Figure 5). PAN_45.7_-*b*-PMMA_20.4_ (Figure 5a) shows a porous structure with domain sizes of about 100 nm. In between the porous network, deeper pores and holes were visible. PAN_45.7_-*b*-PMMA_8.9_ (Figure 5b) shows a surface of a greater roughness with bigger domain sizes and holes. Regarding the SEM images of PAN_58.4_-*b*-PMMA_24.0_ (Figure 5c), lamellar structures are observed after pyrolysis, thus proving the possible soft-templating approach based on the previous self-assembly of the block copolymer domains. During the thermal treatment, the PAN lamellae were stabilized, whereas the PMMA lamella in between were burned during the pyrolysis step, resulting in the PAN lamella being left. This shows that the structure of the stabilized PAN with its lamellar morphology was successfully maintained at high temperatures during the pyrolysis step.

### 3.3. Post-Modification Using 3-Ferrocenyl Propylamine

**Scheme 2:**
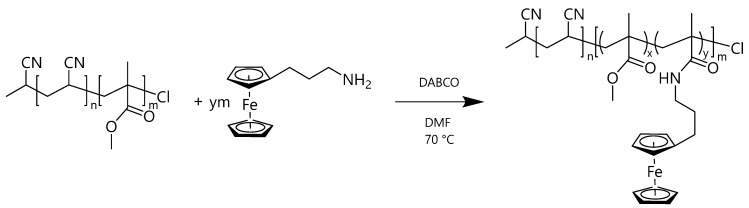
Post-modification of the PAN-*b*-PMMA block copolymer using 3-ferrocenyl propylamine. Abbreviations: 1,4-diazabicyclo [2.2.2]octane (DABCO); dimethyl formamide (DMF).

A metal precursor was incorporated selectively into one block domain to introduce more organic and final ceramic material functionalities. For this purpose, a polymer-analogous reaction to the BCP was chosen by nucleophilic attack at the carbonyl group of the PMMA segment, followed by ceramic conversion. 3-ferrocenyl propylamine was used for the partial amidation of the MMA´s carbonyl moiety. The 3-ferrocenyl propylamine was synthesized according to a previously published route by the group of Su [69]. To synthesize and obtain the desired amine nucleophile, ferrocene carboxaldehyde was first reacted with acetonitrile in an aldol condensation-like reaction. The resulting 2-cyanovinylferrocene was then hydrogenated using a Raney-Ni catalyst and hydrogen. The amidation of the PMMA block segment was performed in DMF in the presence of 1,4-diazabicyclo [2.2.2]octane (DABCO). An orange solid was isolated after precipitation of the polymer and washing (Scheme 2).

**Figure 6:**
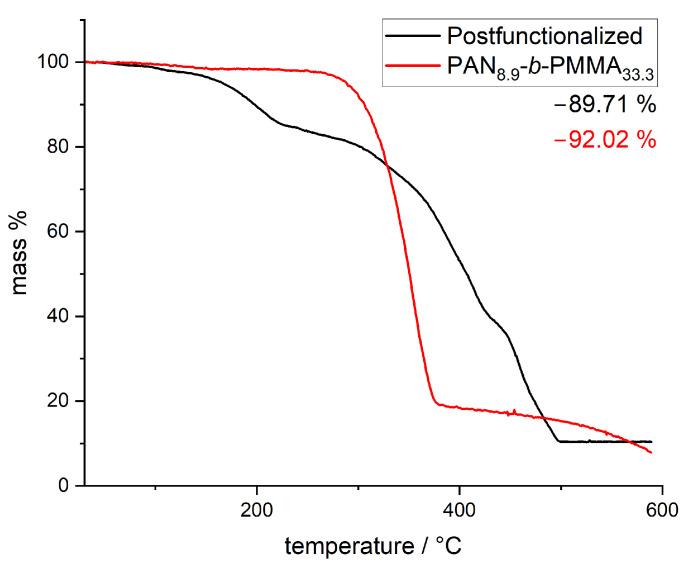
TGA measurements of the unmodified PAN_8.9_-*b*-PMMA_33.3_ (red) and the block copolymer after the partial amidation using 3-ferrocenyl propylamine (black). The measurements were performed under synthetic air from 30 °C to 600 °C with a heating rate of 10 K min^–1^.

The ^1^H-NMR spectrum of the polymer shows the significant aromatic signal of the cyclopentadienyl rings of the ferrocene. In addition, the integration of the PAN and PMMA signals show a change in their ratio before and after the amidation, which indicates that the 3-ferrocenyl propylamine was successfully bound to the polymer (Figure S5). Out of the NMR spectrum signals, the degree of functionalization can be calculated indicating that around 30% of the PMMA underwent the amidation. With this degree of functionalization, the new molar mass of the functionalized polymer can be estimated to be 63 kg mol^–1^ (PAN_8.9_-*b*-P(MMA_23.3_-*co*-(*N*-3-(ferrocenyl)propyl)methacrylamide)_31.0_). SEC measurements of the functionalized polymer showed no significant signal shift compared to the BCP (Figure S8). This can be due to the hydrodynamic volume of the polymer not being influenced by the ferrocene. Analyzing the polymers using the TGA under synthetic air (Figure 6) shows an additional step, indicating a mass loss of approximately 10 wt.-%, which shows that the ferrocene-containing polymer decomposed earlier at about 190 °C. In contrast, the unmodified one stays stable at about 290 °C and decomposes in one step.

Preparing a film out of the functionalized polymer and conducting the same stabilization and pyrolysis as the other films shows an interesting difference in surface composition and surface structure between the stabilized and the pyrolyzed films.

**Figure 7:**
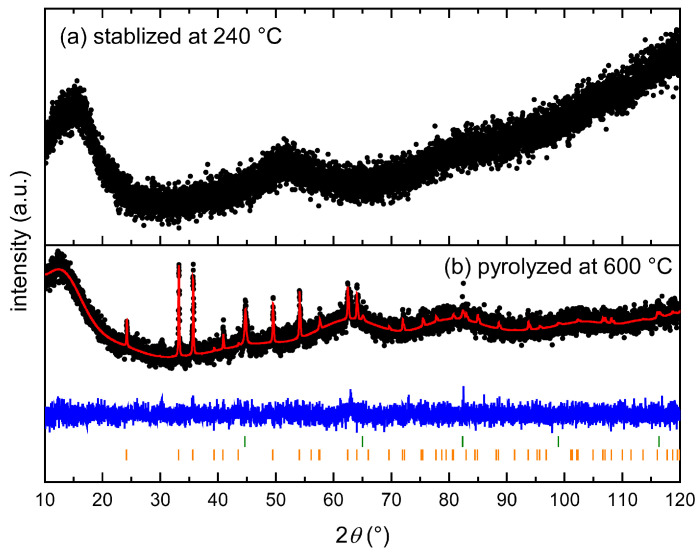
Powder X-ray diffraction patterns of (**a**) the at 240 °C stabilized and (**b**) at 600 °C pyrolyzed film. Black dots represent the experimental data, the red line the fit, the blue line the difference between experiment and fit, and the green and orange ticks the Bragg positions of elemental Fe and Fe_2_O_3_.

To gain structural information on the prepared films, powder X-ray diffraction experiments were conducted on the stabilized and the pyrolyzed films. Figure 7 depicts the results of the experiments. While the stabilized film (Figure 7a) is amorphous, the pyrolyzed sample (Figure 7b) clearly shows the presence of Bragg reflections. These can be attributed to elemental α-Fe (W-type, space group *Fm*$\bar{3}$*m*) [77] and Fe_2_O_3_ (Al_2_O_3_ type, *R*$\bar{3}$*c*) [78]. In addition, a broad signal at 2*θ*~12° is visible, which can potentially be attributed to the carbon content. The lattice parameters deduced from the Rietveld refinements [79,80] (Fe: *a* = 286.64(5) pm; Fe_2_O_3_: *a* = 503.56(4), *c* = 1374.7(2) pm) are in good agreement with the ones reported in the literature [77,78]. The crystallite sizes were determined from the fits to be 25(3) nm for α-Fe and 63(3) nm for Fe_2_O_3_. Finally, 13(2) mass-% α-Fe and 87(2) mass-% Fe_2_O_3_ were deduced, suggesting that initially, a reduction of the Cp_2_Fe moiety to elemental Fe takes place, followed by an oxidation to Fe_2_O_3_.

**Figure 8:**
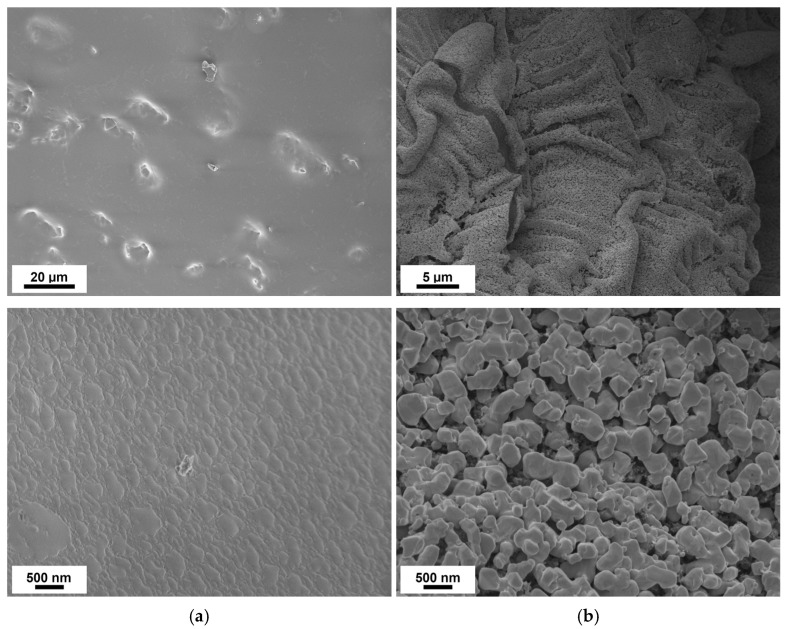
Scanning electron microscopy images of the 3-ferrocenyl propylamine modified BCP after stabilization at 240 °C under synthetic air (**a**) and after pyrolysis at 600 °C under nitrogen atmosphere (**b**).

**Figure 9:**
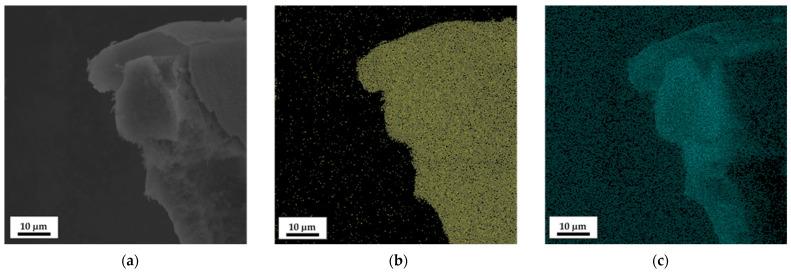
Scanning electron microscopy images of (**a**) the pyrolyzed 3-ferrocenyl propylamine modified BCP and energy dispersive X-ray mapping (**b**,**c**) of iron and oxygen, respectively.

Figure 8 shows the SEM images of the stabilized film (Figure 8a) and the pyrolyzed sample (Figure 8b). After the stabilization, the film shows a closed, rather smooth surface without any pores or holes. At higher magnifications, contours can be observed, which resemble no ordered structures. In contrast, the pyrolyzed sample shows a rough surface with clumped-up crumbs with domain sizes of around 400 nm. This indicates that the pyrolysis step removes the PMMA block from the BCP, resulting in the stabilized PAN being left. The ferrocene bound to the PMMA is converted to the corresponding ceramics comprising different iron oxide species during the stabilization process, despite the stabilization being under a nitrogen atmosphere, as seen in the samples’ XRD spectra. EDX mappings of the pyrolyzed sample were taken to confirm further that the iron from the ferrocene remains in the sample (Figure 9). The iron mapping of the sample (Figure 9b) shows the presence of iron on the surface of the sample, whereas no iron is detected in the background. The oxygen mapping (Figure 9c) further proves the presence of the developed iron oxide.

## 4. Conclusions

The synthesis of polyacrylonitrile-based block copolymers is an advantageous way to produce tailored carbon materials with defined geometries. Within this study, a combination of ARGET-ATRP and standard ATRP was used to synthesize PAN-*b*-PMMA block copolymers, with molecular weights ranging from 44 to 82 kDa, dispersities between 1.19 and 1.5, and volume fractions of PMMA reaching from 0.16 to 0.75, reflecting good control over the combined ARGET-ATRP methods and tailoring the composition of the BCPs. Stabilizing the PAN block under air conditions with the subsequent pyrolysis sacrificing the second block leads to defined porous carbon structures, capable of maintaining morphologies formed by the self-assembly of the BCP, as shown with a lamella structure with a domain size of 47 ± 5 nm. Modifying the sacrificial block before stabilization and pyrolysis is an interesting pathway to alter the ceramic yield, with the ceramic yield referring to the ratio of the mass of the sample before the stabilization and pyrolysis against the mass of the sample after both steps. The designed macromolecular architecture in this work enabled the application of a convenient amidation protocol of the PMMA block with 3-ferrocenyl propylamine, gaining access to PAN-based functional BCPs. To show the potential of the herein-investigated combination of molecular polymer design, post-modification, stabilization, and, finally, ceramization, we established a thermal treatment protocol aiming for functional ceramics based on the soft templating strategy. As a result, carbon-based materials with tailored iron/iron oxide (Fe_2_O_3_) and morphology on the surface were obtained, as confirmed by XRD and SEM-EDX measurements. The herein-shown preparation method can be used to access various macro- and microporous materials exhibiting hierarchical morphologies and functionalities, which can find applications such as catalysts, magnetic materials, sensors, and electrochemical devices.

## Figures and Tables

**Table 1:** SEC data of the polymers were measured against the PMMA standard. The molecular weights of the PAN macroinitiators were determined using PEG standards, as PEG and PAN have a similar hydrodynamic radius [70]. The composition of the block copolymers was calculated using the NMR data. The density of PMMA (1.18 g cm^−3^) and PAN (1.18 g cm^−3^) was used to obtain the volume fraction [71,72].

	M_n_/kg mol^−1^ (vs. PMMA)	*Ð* (vs. PMMA)	φ_PMMA_/% (NMR)
PAN_45.7_	74.4	1.13	/
PAN_11.1_	19.2	1.24	/
PAN_58.4_	80.77	1.34	/
PAN_45.7_-*b*-PMMA*_20_*_.4_	105.9	1.19	31
PAN_45.7_-*b*-PMMA_8.9_	87.4	1.22	16
PAN_11.1_-*b*-PMMA_33.3_	53.4	1.50	75
PAN_58.4_-*b*-PMMA_24.0_	152.8	1.38	43

## Data Availability

All relevant data are implemented into the manuscript. Additional data can be found in the supporting information or upon request.

## Supplementary Materials

The following supporting information can be downloaded at: https://www.mdpi.com/article/10.3390/polym16152142/s1, Figure S1. SEC measurements of the PAN macroinitiators. Measurements were performed using DMF with LiBr (1 g L^−1^) as the mobile phase using PEG standards.; Table S1. Overview of the PAN macroinitiator syntheses.; Table S2. Overview of the PAN_58.4_ macroinitiator synthesis.; Table S3. SEC data of the PAN macroinitiators measured against PEG standards.; Table S4. Overview of the PAN-*b*-PMMA block copolymer syntheses.; Figure S2. ^1^H-NMR spectrum of PAN_45.7_-*b*-PMMA_8.9_ in DMSO-d_6_.; Figure S3. ^1^H-NMR spectrum of PAN_58.4_-*b*-PMMA_20.0_ in DMSO-d_6_.; Figure S4. ^1^H-NMR spectrum of PAN_11.1_-*b*-PMMA_33.3_ in DMSO-d_6_.; Figure S5. ^1^H-NMR spectrum PAN_11.1_-*b*-PMMA_33.3_ after post-modification using 3-ferrocenyl propylamine, measured in DMSO-d_6_.; Figure S6. SEC measurements of PAN_58.4_ (red) and PAN_58.4_-*b*-PMMA_20.0_ (black). DMF with LiBr 1 g L^−1^ was used as the mobile phase, using PMMA standards.; Figure S7. DSC measurements of the polymers under nitrogen atmosphere.; Figure S8. SEC measurements of PAN_8.3_-*b*-PMMA_33.3_ (red) and the polymer after the post-functionalization with 3-ferrocenyl propylamine (black). DMF with LiBr 1 g L^−1^ was used as the mobile phase, using PMMA standards.; Figure S9. DSC measurements of PAN_11.1_-*b*-PMMA_33.3_ before the amidation (black) and after the partial amidation with 3-ferrocenyl propylamine (red). Measurements were performed under a nitrogen atmosphere.; Figure S10. Stabilization run of PAN_11.1_-*b*-PMMA_33.3_ at 240 °C for 10n hours under synthetic air.; Figure S11. Pyrolysis run of the post-functionalized PAN_11.1_-*b*-PMMA_33.3_ at 600 °C for 5 h under a nitrogen atmosphere.; Figure S12. SEM images of the films of (a) PAN_8.9_-bPMMA_33.3_ and (b) PAN_8.9_-bPMMA_33.3_ after the ferrocene post-modification.; Figure S13. TGA measurements of the unstabilized PAN_45.7_-b-PMMA_20.4_ from 30 °C to 600 °C under nitrogen atmosphere (black) compared with the pyrolysis run of the stabilized sample (red).; Figure S14. Transmission electron microscopy images of thin films of PAN_11.1_-*b*PMMA_33.3_, with the darker domains being the PAN and the lighter domains the PMMA. The size of the lamella (dark + light) were measured to be 47 ± 5 nm.
