# Supplementary material for: Ferrocene-Modified Polyacrylonitrile-Containing Block Copolymers as Preceramic Materials
Source: Polymers (Basel). 2024 Jul 28;16(15):2142. doi: 10.3390/polym16152142 (PMC11314306; doi:10.3390/polym16152142)
Supplement: Supplementary file 1 [file polymers-16-02142-s001.zip › polymers-3106749-supplementary.pdf]

Supporting Information for

Ferrocene-Modified Polyacrylonitrile-Containing Block Copolymers as Preceramic Materials

Sebastian Heinz [1,†], Lea Gemmer [1,†], Oliver Janka [2] and Markus Gallei [1,3,*]

[1] Polymer Chemistry, Campus C4 2, Saarland University, 66123 Saarbrücken, Germany; sebastian.heinz@uni-saarland.de (S.H.); lea.gemmer@tu-darmstadt.de (L.G.)
[2] Inorganic Solid State Chemistry, Campus C4 1, Saarland University, 66123 Saarbrücken, Germany; oliver.janka@uni-saarland.de
[3] Saarene, Campus C4 2, Saarland Center for Energy Materials and Sustainability, 66123 Saarbrücken, Germany
* Correspondence: markus.gallei@uni-saarland.de
† These authors contributed equally to this work.

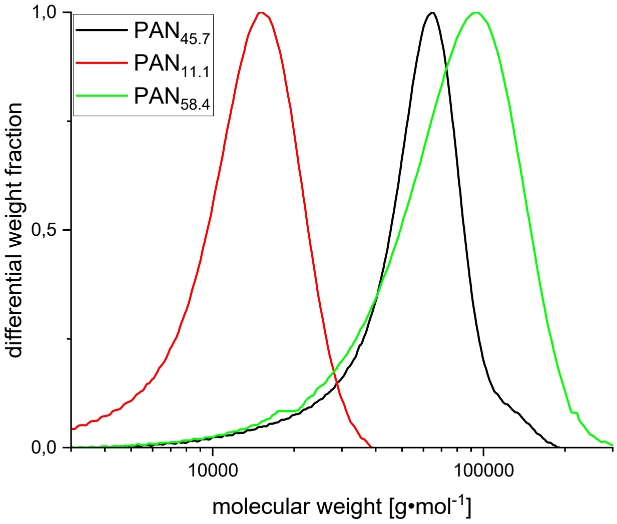

Figure S1. SEC measurements of the PAN macroinitiators. Measurements were performed using DMF with LiBr (1 g L^{-1}) as the mobile phase using PEG standards.

Table S1. Overview of the PAN macroinitiator syntheses.

		$PAN_{45.7}$	$PAN_{11.1}$	$PAN_{58.4}$
AN	eq.	1500	800	4000
	n / mmol	137.4	200.0	38.2
	V / mL	9.0	13.3	2.5
$Cu^{II}(TPMA)Cl_2$ (0.2 M)	eq.	0.05	0.05	0.2
	n / µmol	4.6	12.5	1.9
	V / µL	22.9	62.5	12.73
BPN (0.5 M)	eq.	1	1	1
	n / µmol	91.6	250.0	9.6
	V / µL	183.2	500.0	6.37
$Sn(EH)_2$/TPMA (0.1 M)	eq.	0.5	0.5	2
	n / µmol	45.8	125.0	20
	V / µL	458.0	1250.0	191
Ethylene carbonate V / mL		21.6	31.9	6
Reaction time / h		16.5	6	40

Table S2. Overview of the $PAN_{58.4}$ macroinitiator synthesis.

		$PAN_{58.4}$
AN	eq.	4000
	n / mmol	38.2
	V / mL	2.5
$Cu^{II}(TPMA)Cl_2$ (0.15 M)	eq.	0.2
	n / µmol	1.9
	V / µL	12.73
CPN (1.5 M)	eq.	1
	n / µmol	9.6
	V / µL	6.37
$Sn(EH)_2$/TPMA (0.1 M)	eq.	2
	n / µmol	20
	V / µL	191
DMSO V / mL		6
Reaction time / h		40

Table S3. SEC data of the PAN macroinitiators measured against PEG standards.

	M_n / kg mol^{-1}	*Đ*
$PAN_{45.7}$	45.7	1.32
$PAN_{11.1}$	11.1	1.29
$PAN_{58.4}$	58.4	1.48

Table S4. Overview of the PAN-*b*-PMMA block copolymer syntheses.

		$PAN_{45.7}$-*b*-$PMMA_{20.4}$	$PAN_{11.1}$-*b*-$PMMA_{8.9}$	$PAN_{11.1}$-*b*-$PMMA_{33.3}$		$PAN_{58.4}$-*b*-$PMMA_{24.0}$
macro initiator	Initiator	$PAN_{45.7}$	$PAN_{45.7}$	$PAN_{11.1}$		$PAN_{58.4}$
	eq.	1	1	1		1
	n / µmol	4.1	4.0	31.5		1.8
	m / mg	189.0	185.0	350.0		105.5
Cu^{I}(bpy)Cl (0,2 M)	eq.	1	1	1	Cu^{I}(TMEDA)Cl (0.25 M)	2.5
	n / µmol	4.1	4.0	31.5		9.4
	V / µL	20.5	20.2	157.5		49.7
MMA	eq.	1430	2000	1300		3000
	n / mmol	5.9	8.0	41.0		11.3
	V / mL	0.63	0.85	4.36		1.2
DMSO V / mL		6.0	6.5	32.1		9.6
reaction time / h		22.25	44	64		61

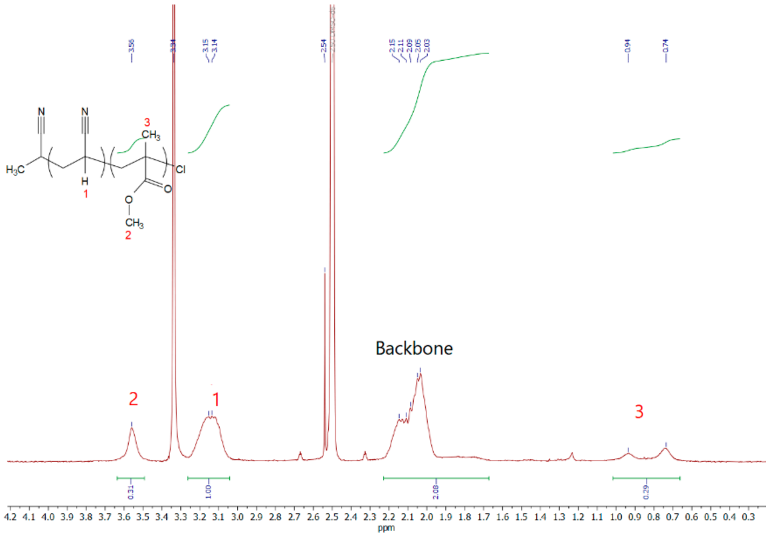

Figure S2. ^{1}H-NMR spectrum of $PAN_{45.7}$-*b*-$PMMA_{8.9}$ in DMSO-d_6.

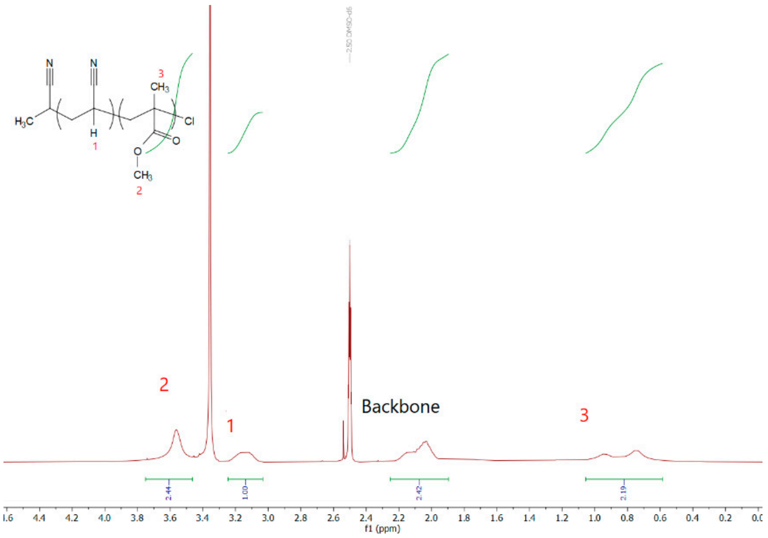

Figure S3. ^{1}H-NMR spectrum of $PAN_{58.4}$-*b*-$PMMA_{20.0}$ in DMSO-d_6.

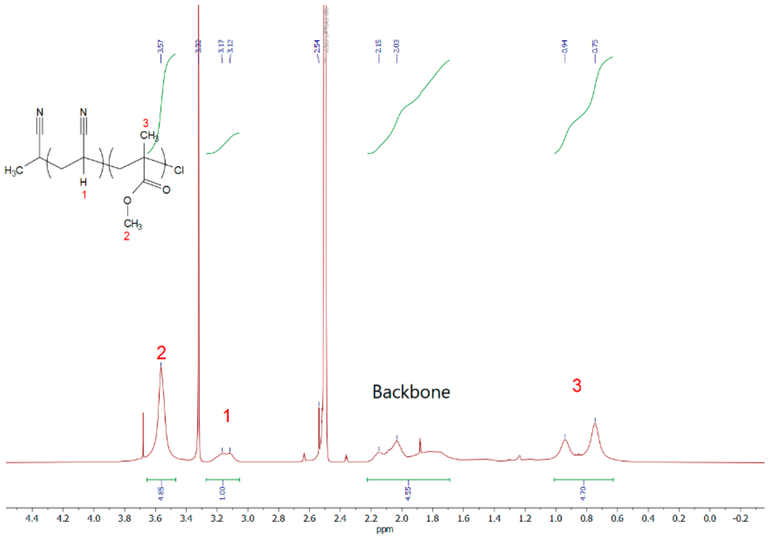

Figure S4. ^{1}H-NMR spectrum of $PAN_{11.1}$-*b*-$PMMA_{33.3}$ in DMSO-d_6.

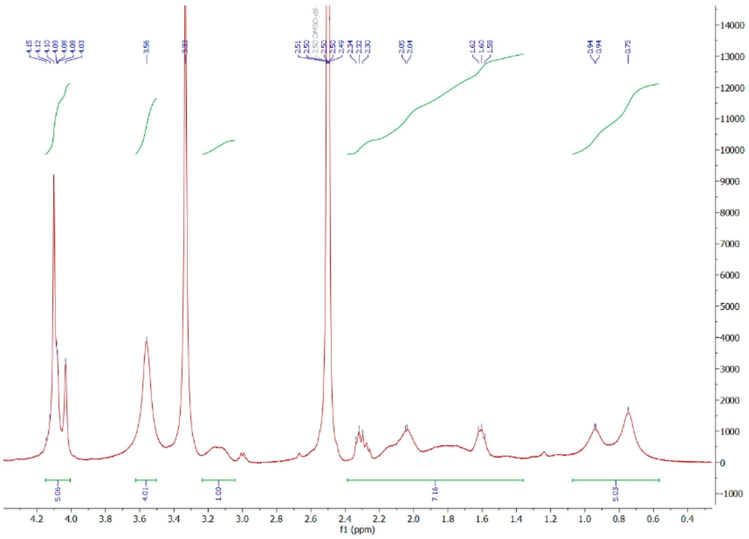

Figure S5. ^{1}H-NMR spectrum $PAN_{11.1}$-*b*-$PMMA_{33.3}$ after post-modification using 3-ferrocenyl propylamine, measured in DMSO-d_6.

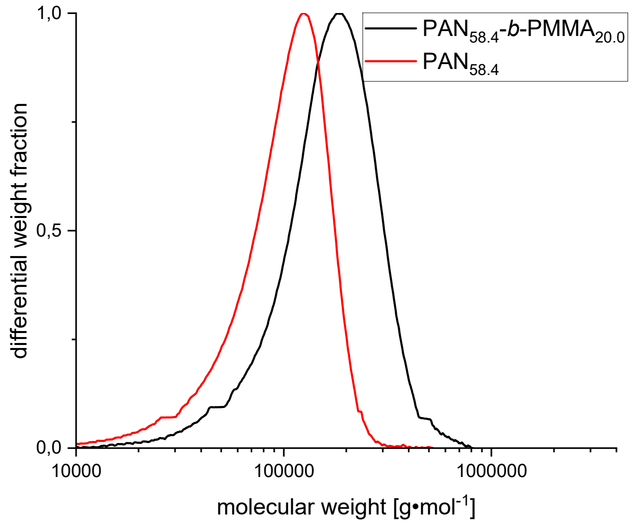

Figure S6. SEC measurements of $PAN_{58.4}$ (red) and $PAN_{58.4}$-*b*-$PMMA_{20.0}$ (black). DMF with LiBr 1 g L^{-1} was used as the mobile phase, using PMMA standards.

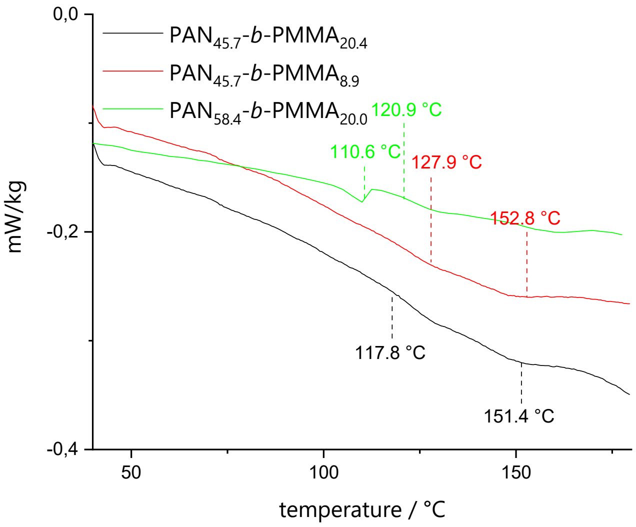

Figure S7. DSC measurements of the polymers under nitrogen atmosphere.

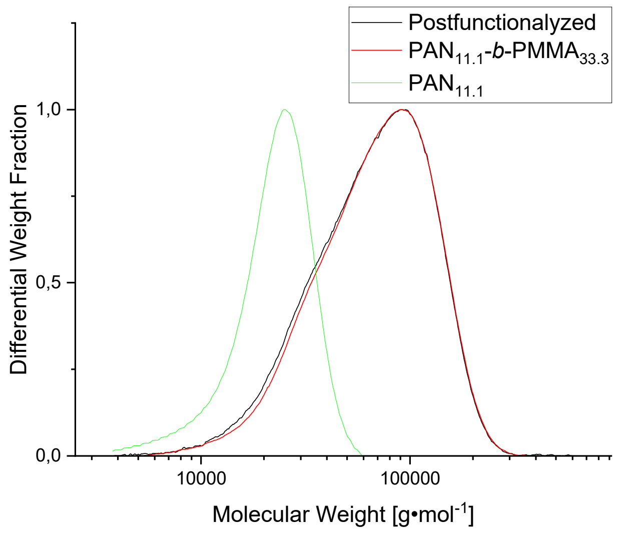

Figure S8. SEC measurements of PAN8.3-*b*-PMMA33.3 (red) and the polymer after the post-functionalization with 3-ferrocenyl propylamine (black). DMF with LiBr 1 g L^{-1} was used as the mobile phase, using PMMA standards.

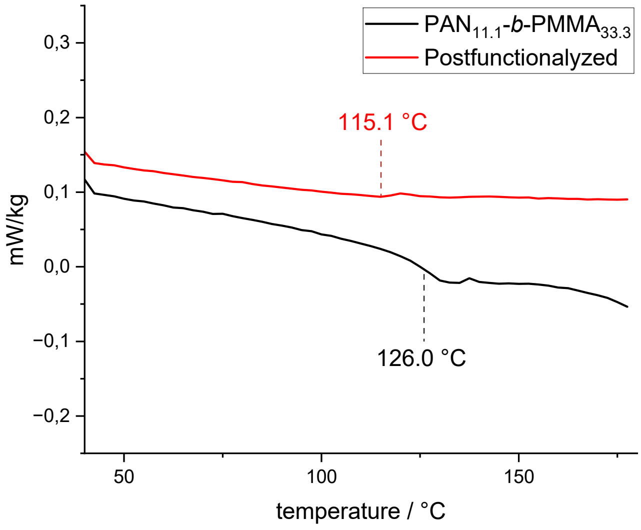

Figure S9. DSC measurements of PAN11.1-*b*-PMMA33.3 before the amidation (black) and after the partial amidation with 3-ferrocenyl propylamine (red). Measurements were done under a nitrogen atmosphere.

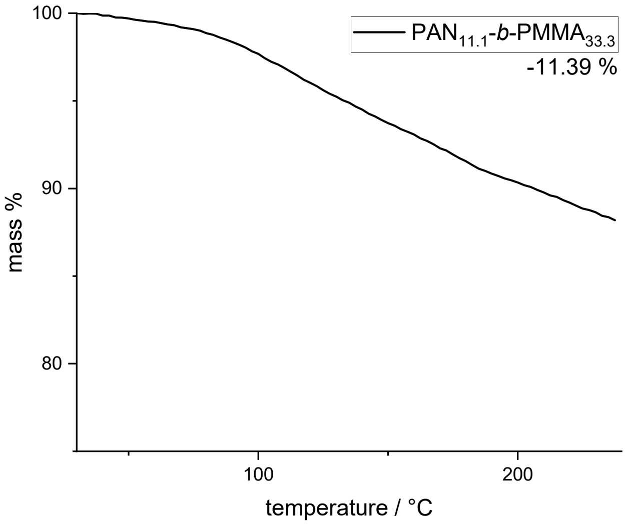

Figure S10. Stabilization run of PAN$_{11.1}$-*b*-PMMA$_{33.3}$ at 240 °C for 10n hours under synthetic air.

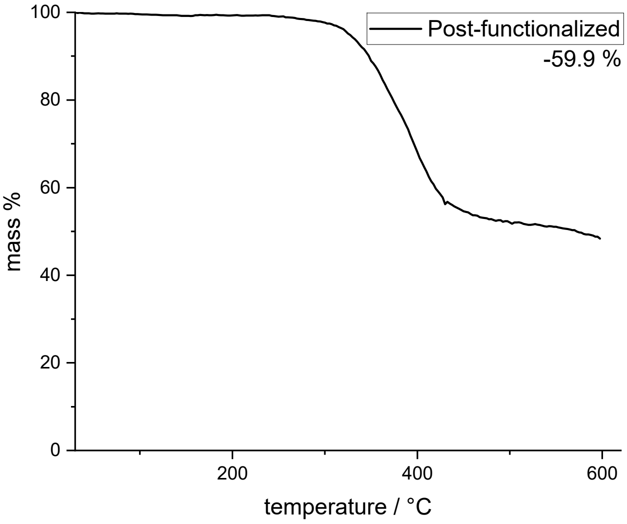

Figure S11. Pyrolysis run of the post-functionalized PAN$_{11.1}$-*b*-PMMA$_{33.3}$ at 600 °C for 5 hours under a nitrogen atmosphere.

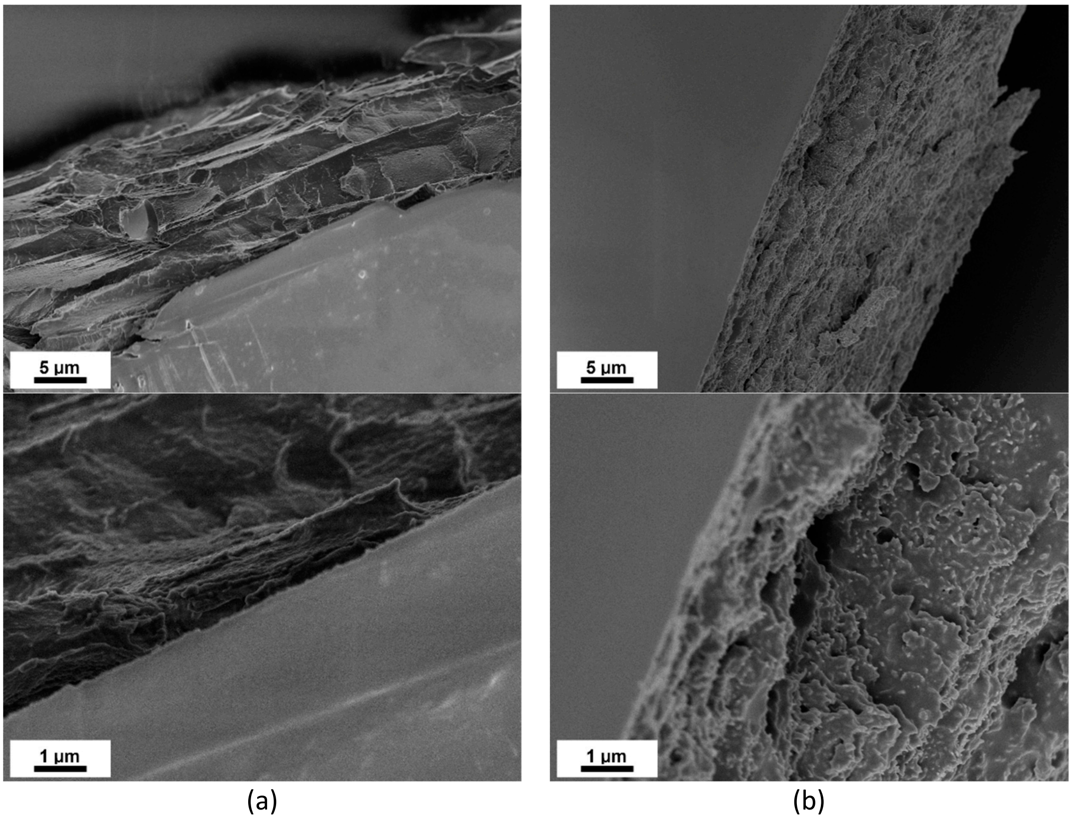

Figure S12. SEM images of the films of (a) $PAN_{8.9}$-b$PMMA_{33.3}$ and (b) $PAN_{8.9}$-b$PMMA_{33.3}$ after the ferrocene postmodification.

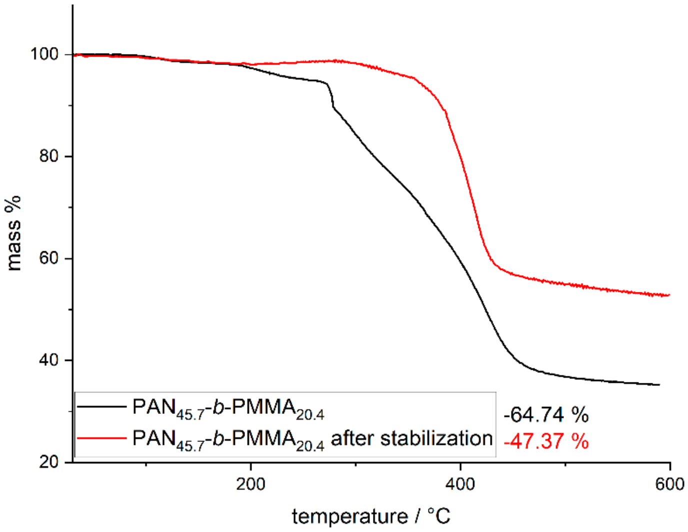

Figure S13. TGA measurements of the unstabilized $PAN_{45.7}$-b-$PMMA_{20.4}$ from 30 °C to 600 °C under nitrogen atmosphere (black) compared with the pyrolysis run of the stabilized sample (red).

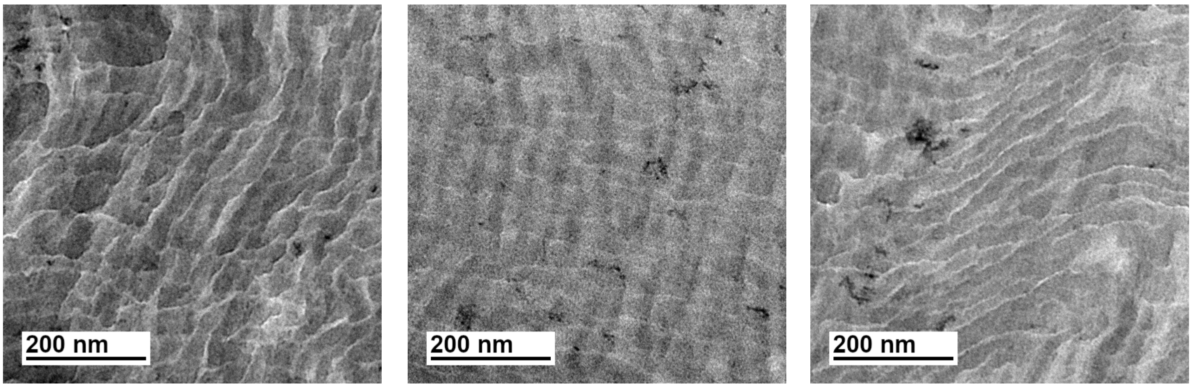

Figure S14. Transmission electron microscopy images of thin films of $PAN_{11.1}$-*b*$PMMA_{33.3}$, with the darker domains being the PAN and the lighter domains the PMMA. The size of the lamella (dark+light) were measured to be 47±5 nm.
